# Fixed Cut-Off for FEV1/FEV6 and FEV6 in Detection of Obstructive and Restrictive Patterns

**DOI:** 10.5812/ircmj.8163

**Published:** 2013-02-05

**Authors:** Rokhsareh Aghili, Maryam Kia, Alipasha Meysamie, Seyed Mojtaba Aghili, Omalbanin Paknejad

**Affiliations:** 1 Endocrine Research Center (Firouzgar), Institute of Endocrinology and Metabolism (Hemmat Campus), Tehran University of Medical Sciences, Tehran, IR Iran; 2 Department of Internal medicine, Tehran University of Medical Sciences, Tehran, IR Iran; 3 Department of Community and Preventive Medicine, Faculty of Medicine, Tehran University of Medical Sciences, Tehran, IR Iran; 4 Department of Emergency Medicine, Imam Khomeini Hospital, Tehran University of Medical Sciences, Tehran, IR Iran; 5 Division of Pulmonology, Shariati Hospital, Tehran University of Medical Sciences, Tehran, IR Iran

**Keywords:** Pulmonary Disease, Chronic Obstructive, Pulmonary Function Tests

## Abstract

**Background:**

Chronic obstructive pulmonary diseases (COPD) have been defined by the Global Initiative for Chronic Obstructive Lung Disease (GOLD) as irreversible conditions which are diagnosed by fixed cut-off points of FEV1/FVC.

**Objective:**

The aim of this study was to determine the cut-off points for FEV1/FEV6 ratio and FEV6 as alternatives for FEV1/FVC and FVC in detection of airway obstruction and lung restriction, respectively.

**Materials and Methods:**

A total of 318 Spiro metric examinations of subjects referred to Shariati hospital were analyzed. A subject was considered to have obstruction if FEV1/FVC was lower than 70%. The restriction was defined as FVC < 80% in the absence of obstruction. The Sensitivity, specificity, positive predictive value (PPV) and negative predictive value (NPV) of FEV1/FEV6 and FEV6 were calculated.

**Results:**

This study shows that the current cut-off points used to detect obstruction and restriction can be replaced by FEV1/FEV6 < 71% and FEV6 < 83%, respectively. FEV1/FEV6 had sensitivity of 95.5% and specificity of 99.4%; the PPV and NPVs were 99.3% and 96.3%. The prevalence of obstruction was 49.4%. For restrictive pattern, FEV6 had sensitivity of 93%, specificity of 79.5% with PPV of 18% and NPV of 99.5%. The prevalence of restriction was 6.3%.

**Conclusions:**

The FEV1/FEV6 ratio can be used as a valid surrogate for FEV1/FVC in the diagnosis of airway obstruction, especially for screening purposes in high-risk populations for COPD. Moreover, FEV6 is an acceptable alternative for FVC in detection of restrictive pattern.

## 1. Background

Chronic obstructive pulmonary diseases (COPD) have been defined by the Global Initiative for Chronic Obstructive Lung Disease (GOLD) as irreversible conditions which are diagnosed by fixed cut-off points of FEV1/FVC < 70% (1, 2). In addition, FVC < 80% in a normal FEV1/FVC ratio is considered as restrictive pulmonary disease (3). Spirometer is the most common test for evaluating pulmonary function (3) which yields the above-mentioned variables (FEV1/FVC, FVC) (4-7); hence, its significance in the initial screening of COPD is emphasized (8-11). To reach an acceptable and logical FVC, two criteria have been recommended for the end of the test:

1. Subjects cannot or should not continue exhalation.

2. The volume-time curve should remain constant for at least one second (1 ≤) (less than 0.025l), and subjects (≥ 10 years) should continue exhaling for 6 seconds or more (In children under 10 years this duration should be 3 seconds or more). In the elderly, or those with obstructive pulmonary disease, exhalation should exceed 6 seconds (up to 15 seconds), and if the curve does not touch the base, the technician should encourage the patient to reach the end of test criteria (12). Therefore, unacceptable FVCs are rather common, and in some cases they are ignored because of time constraints. If the physician oversees the absence of the end of test curve, or the short duration of exhalation, the test may be interpreted solely on the bases of the reported figures (13). Growing evidence shows that reducing the exhalation maneuver to 6 seconds (FEV6) can be a suitable substitute for FVC in the FEV1/FVC ratio (13-17). The advantages of FEV6 are as follows:

Ease in performance, both for the subject and technician (3, 18)

Removing the limitations of accuracy in detecting very low flows at the end of the maneuvers

Reducing the duration of the spirometer

Reducing the complications of spirometer, such as syncope (19-21)

## 2. Objectives

The objective of the present study was to find the best cut-off points for FEV1/FEV6 and FEV6 in the diagnosis of obstructive and restrictive pulmonary diseases.

## 3. Materials and Methods

The population under study consisted of subjects referred to the spirometer unit of Shariati hospital and in whom spirometer was not contraindicated. Informed consent was obtained from those eligible subjects who desired to participate in the study. The subjects were included in the study up to the point where sample size was completed. The spirometers were performed by experienced technicians with the spirometer model “Viasys Health, Master Scope version 4.6” (Care, Hoechberg, Germany). Variables such as FEV1/FEV6, FEV6, FEV1/FVC, and FVC were measured and compared with the lower limit normal (LLN) values that have been specified in the NHANES III study (22). In patients in whom the diagnosis of COPD was considered according to the GOLD criteria (FEV1/FVC < 70%), using the ROC curve, the value of FEV1/FEV6 was determined for the highest collective sensitivity and specificity. Moreover, in individuals whom the FEV1/FVC ratio was normal and FVC < 80% (restrictive pulmonary diseases) the best cut-off value for FEV6 was calculated by using the ROC curve. PPV and NPVs were calculated for both parameters.

### 3.1. Data Analysis

Data were analyzed with SPSS 18. The best cut-off values were calculated for the variables using the ROC curve. The sensitivity and specificity were calculated using contingency tables. In addition, positive predictive values and negative predictive values were calculated for both indices.

## 4. Results

318 spirometers were studied; 107 (33.6%) were female and 211 (66.4%) were male. Their age ranged from 17-87. The mean age was 52 (± 14.7). The baseline characteristics of the study population have been illustrated in Table 1. GOLD criteria were used to detect patients with restrictive pulmonary disease in the spirometer. Based on the GOLD guideline and the degree of pulmonary involvement, these patients were classified into four subgroups (1):

Stage 1: FEV1/FVC < 70% and FEV1 ≥ 80%

Stage 2: FEV1/FVC < 70% and 50% ≤ FEV1 < 80%

Stage 3: FEV1/FVC < 70% and 30% ≤ FEV1 < 50%

Stage 4: FEV1/FVC < 70% and FEV1 < 30

**Table 1.** Patients’ Characteristics

Gender	Number	Age, y, Mean ± SD	Height, cm, Mean ± SD	Weight, kg, Mean ± SD	Not Obstructed No. (%)		Obstructed No. (%)				
					Normal	Restricted	Stage 1	Stage 2	Stage 3	Stage 4	Total
**Male**	211	54.2 ± 14.5	169.2 ± 6.6	75.7 ± 14.8	91 (28/6)	14 (4.4)	17 (5.3)	50 (15.7)	31 (9.7)	8 (2.5)	106 (33.3)
**Female**	107	47.4 ± 13.9	158.4 ± 5.6	73.2 ± 15.0	50 (15.7)	6 (1.9)	12 (3.8)	23 (7.3)	13 (4.1)	3 (1)	51 (16.1)
**Total**	318	51.9 ± 14.7	165.6 ± 8.1	74.9 ± 15.0	141 (44.3)	20 (6.3)	29 (9.1)	73 (23)	44 (13.8)	11 (3.5)	157 (49.4)

**Figure 1.**
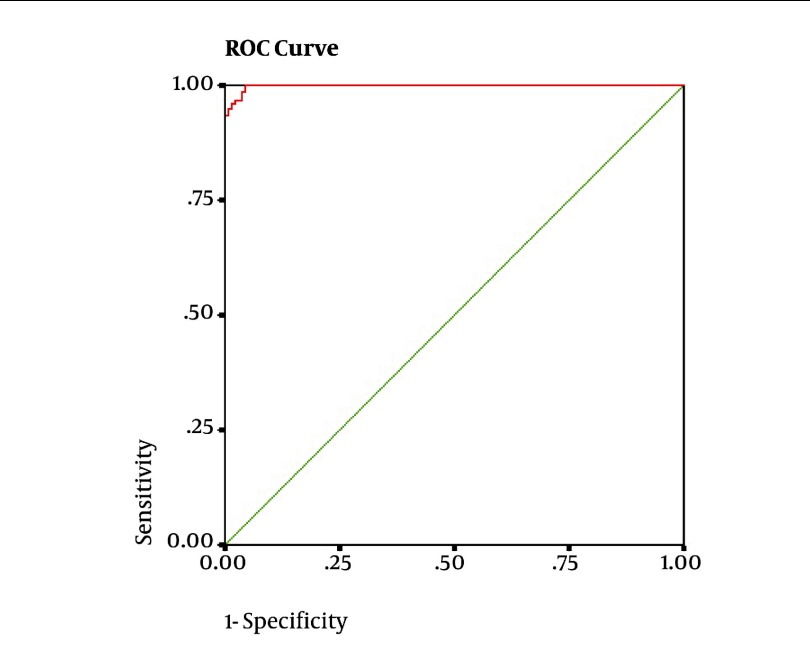
The ROC curve obtained for FEV1/FEV6, using the GOLD standard of FEV1/FVC < 70% for detection of pulmonary obstructive disease

**Figure 2.**
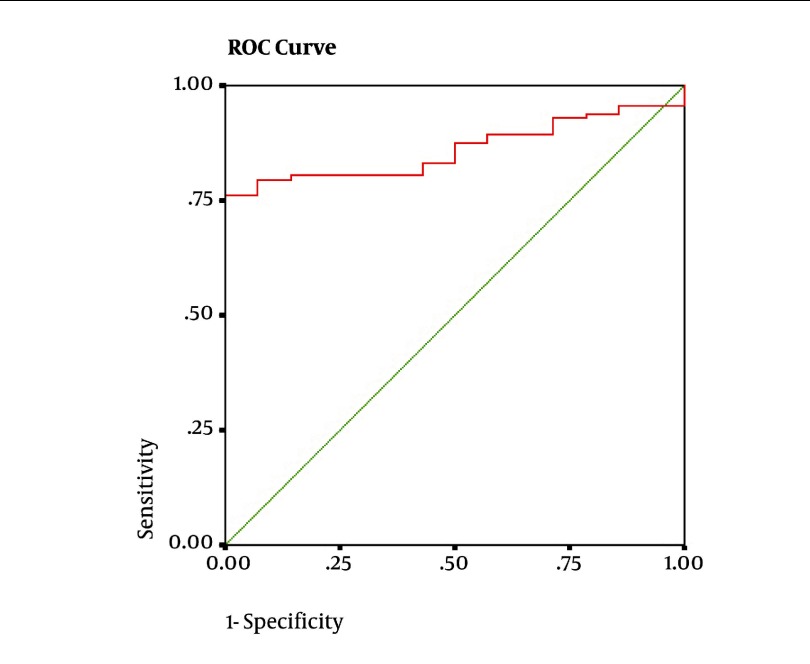
The ROC curve obtained for FEV6 using the GOLD standard of FVC < 80% for detection of pulmonary obstructive disease

Detection of pulmonary obstructive disease on the basis of spirometer results: (Figure 1). The FEV1/FEV6 cut-off point for detection was 71%. The sensitivity and specificity of FEV1/FEV6 were 95.5% and 99.4% respectively; the PPV and NPVs were reported to be 99.3% and 96.3%. The diagnostic accuracy was 97.5%. The prevalence of obstruction in the study sample was 49.4% (157 out of 318). The findings have been illustrated in Table 2. The discordant cases were 11 in our study. The values obtained for FEV1/FVC and FEV1/FEV6 were very close to the determined cut-off points. The detection of restrictive pulmonary disease on the basis of spirometer results: (Figure 2). FEV6 was evaluated as a substitute for FVC in the restrictive pulmonary disease in subjects whose FEV1/FVC ratio was normal. Upon analysis of the ROC curve, the obtained cut-off point for FEV6 was 83%. The sensitivity and specificity of FEV6 were 93% and 79.5%respectively; the PPV and NPVs were 18% and 99.5%. The accuracy of this method was 80%. The prevalence of restrictive disease was 6.3% (20 out of 318). The results have been presented in Table 3. The discordant cases were 2 in the current study. The values obtained for FEV6 and FVC were very close to the cut-off points determined.

**Table 2.** Comparison of FEV1/FEV6 with FEV1/FVC in Detection of Pulmonary Obstructive Disease

FEV1/FEV6	FEV1/FVC		Total
	Obstruction, No. (%)	No obstruction, No. (%)	
**Obstruction ( < 73%), No. (%)**	146 (93.6)	1 (0.61)	147
**No obstruction ( > = 73%), No. (%)**	10 (6.4)	161 (99.39)	171
**Total**	156 (100)	162 (100)	318

**Table 3.** Comparison of FVC with FEV6 in Detection of Pulmonary Restrictive Diseases

FEV1/FEV6	FVC		Total
	Restriction, No. (%)	No Restriction, No. (%)	
**Restriction ( < 83%)**	18 (90)	0	18
**No Restriction ( > = 83%)**	2 (10)	141 (100)	143
**Total**	20 (100)	141 (100)	161

## 5. Discussion

Multiple studies have shown that FEV6 is a suitable surrogate for FVC in detection of the restrictive and obstructive pulmonary diseases using the LLN values in NHANES III study (16, 21, 23). The most important objective of this study is to determine the best values of FEV6 and FEV1/FEV6 ratio on the basis of the ROC curve in detection of the restrictive and obstructive pulmonary diseases in lieu of FVC and FEV1/FVC, respectively. The detection of pulmonary obstructive disease on the basis of spirometer results: Our results showed that the prevalence of the obstructive pulmonary disease is 49.4% in the population under study. This figure overlaps with the COPD prevalence reported in the population at-risk (people over 45 years, cigarette smokers, and those having pulmonary symptoms) that is 30-50% (3). However, cut-off points should be used with caution, because the indicators of spirometer are greatly influenced by demographic variables such as age, sex, height and race. It is noteworthy that similar studies need to be conducted using NHANES III to determine the LLN. As we lack the relevant required data in Iran, we used the LLN values obtained from the NHANES III study. The cut-off point obtained for FEV1/FEV6 in our study was 71%. This value was 73% in a similar study performed by Vandevoorde et al. in 2006 (3). The prevalence of the obstructive disease in the present study was 49.4% (157 out of 318); this number was 45.9% in Vandevoorde et al. study. The sensitivity, specificity, positive and negative predictive values have been reported higher than 90% in both studies. Although the cut-off points are very close to each other in these two studies and they are widely used in order to simplify detection of disease, it is possible that the classification is incorrect. Hardie et al. study showed the age-associated reduction in FEV1/FVC and FEV1/FEV6 ratios may result in a false increase in the obstructive pulmonary disease diagnosis (24). In other words, the determined cut-off points are best applied in middle-aged persons. Eventually, despite the method used to determine the disorder; the measured values which are close to the threshold must be interpreted with caution, due to different reasons such as:

1. Spiro metric indicators change during 24 hours (25)

2. Repeatability criteria between two maneuvers recognizes a difference of 150 cc between maximum values of FEV1/FVC as acceptable (12)

3. The coefficient of FEV1 and FVC changes in people with obstructive disease is almost twice that of ordinary people (26)

In another study conducted by Melbye et al. on 3874 acceptable spirometers performed on people ≥ 60 years, using the ROC curve, the best FEV1/FEV6 ratio determined to replace FEV1/FVC < 70% was 73% (18). The populations in our study and Vandevoorde’s were subjects referred from medical centers, while those examined in Melbye’s study were chosen from a homogenous population in a north Norwegian city. In a comparison between FEV1/FEV6 and FEV1/FVC, Rosa et al. performed a study on 40 years and older people in Sao Paulo and found that FEV1/FEV6 can be a suitable substitute for FEV1/FVC in detection of the obstructive diseases, and based on a FEV1/FVC < 0.7, the best cut-off point obtained for FEV1/FEV6 was 75% (27). The strength of this study was the randomization of study population, and the use of reference values (LLN) extracted from the population. Despite the connection between the two aforementioned parameters, in clinical practice, there is always the possibility of discordant cases even for the best cut-off points, and in our study, this figure was very low (3.45%). The detection of the restrictive pulmonary disease on the basis of spirometer results: In the present study, the cut-off point obtained for FEV6 was 83%, that can substitute FVC < 80% in detecting the restrictive pulmonary disease. Restrictive lung disorders are associated with reduced total lung capacity (TLC), while a reduced FVC with normal FEV1/FVC can only suggest the possibility and not the diagnosis of restrictive disease (25). A study conducted by Swanney et al. showed that spirometer algorithms cannot foresee TLC accurately, but it is widely applied in detecting restrictive disorders. They also showed that if the LLN calculated in the NHANES III study is used, FEV6 would be equivalent to FVC (21). In the study conducted by Vandevoorde et al. the prevalence of the restrictive pulmonary disease and NPV were reported as 14.9% and 99.3% respectively. These rates are appropriate for detecting restrictive pulmonary disease using FEV6. However, the foreseen values should be closely examined which can lead to an increased detection of the restrictive patterns in the elderly. In our study, the prevalence of the restrictive pulmonary disease was low (6.3%), and the PPV was reported to be 18%.
